# Measuring the medication literacy level of community residents: a cross-sectional study

**DOI:** 10.3389/fpubh.2025.1605296

**Published:** 2025-08-18

**Authors:** Lixing Huang, Fenfang Wei, Yangjun Liu, Jialin Xu, Jianru Wu, Qian Wang, Shuling Wang, Wenyu Wu

**Affiliations:** ^1^School of Business Administration, Shenyang Pharmaceutical University, Shenyang, Liaoning, China; ^2^Shenzhen Institute of Pharmacovigilance and Risk Management, Shenzhen, Guangdong, China

**Keywords:** medication literacy, community residents, knowledge-attitude-practice (KAP), medication skills, influencing factors, structural equation model

## Abstract

**Objective:**

To assess the current status and identify factors influencing the medication literacy level of community residents, providing a scientific basis to enhance medication literacy and effectively promote the safe use of medications.

**Methods:**

A questionnaire survey was conducted among 2,008 community residents in Shenzhen, employing economic stratification and proportionate sampling methods. The analysis utilized various statistical methodologies, including T-tests, F-tests, multiple linear stepwise regression, and structural equation modeling to assess the current medication literacy status and the factors influencing it.

**Results:**

The levels of medication literacy among community residents typically exhibit a normal distribution. Specifically, 10.16% of residents achieved the excellent level, 40.49% reached the good level, 37.40% were classified as passing, and 11.95% were deemed to have failed. The analysis of factors influencing medication literacy revealed that demographic characteristics, such as age and educational level, as well as various elements of medication knowledge, attitude, behaviors, and skills, significantly impact medication literacy. Notably, medication knowledge emerged as the most critical factor. Structural equation modeling demonstrated that medication knowledge, attitude, behaviors, and skills mediate medication literacy, which can indirectly affect medication literacy and be utilized in multiple ways to improve it and ensure the safety of medication use effectively.

**Conclusion:**

The level of medication literacy among community residents was commendable. However, the proportion of individuals with high medication literacy levels remained insufficient. Key influencing factors, such as medication knowledge, attitude, behaviors, and skills, are explored, offering insights for the government to implement initiatives that publicize medication knowledge, monitor and improve public medication behaviors, promote health education, and enhance community medication literacy and health development.

## 1 Introduction

Medication safety has increasingly garnered the attention of healthcare professionals and researchers within the public health community (1). According to the report of the World Health Organization, improper use of medication accounts for one-third of total deaths worldwide (2). Additionally, the 2023 national health literacy monitoring data revealed that essential medication literacy—encompassing the rational and safe use of medications—was only 28.84%. This figure is notably lower than the overall health literacy rate of 29.70% among Chinese residents (3), underscoring a pressing concern regarding the inadequacy of medication literacy among community residents.

Medication literacy constitutes an essential part of health literacy (4), which primarily refers to the ability of individuals to obtain, evaluate, compute, and comprehend fundamental information about medication and pharmacy-related services to make appropriate decisions related to medication, regardless of the mode of delivery (e.g., written, oral, visual images and symbols) (5). Medication literacy refers to the ability of residents to use medications safely (6, 7). Medication literacy is a significant predictor of rational medication use, encompassing four primary dimensions: medication knowledge, behaviors, skills, and attitudes, which significantly influence the effective management of medication regimens and the development of individualized medication behaviors in patients (8–10).

Currently, there is a lack of assessment tools for medication literacy in the field of public health that target the general population, with most existing tools focusing on patient demographics or chronic diseases. Pantuzza has proposed a conceptual model of medication literacy that reflects a Brazilian perspective, which consists of four fundamental components: functional literacy, critical literacy, communicative literacy, and numerical ability (11). Furthermore, Suka M developed a 14-item Health Literacy Scale (HLS14) designed explicitly for Japanese adults, emphasizing functional, communicative, and critical health literacy (12). Leixiao Li developed a universal medication literacy scale suitable for Chinese university students based on Suka M’s research to investigate medication literacy among Chinese university students (13). Maniaci developed a medication literacy survey for hospitalized patients (14), which Feng Zheng later adapted into Chinese and tested for reliability and validity (15). Shi formulated the Chinese Medication Literacy Scale for Hypertensive Patients (CMLSHP), targeting four dimensions: knowledge, attitudes, skills, and behaviors (16). Sauceda developed medication literacy assessment scales in Spanish and English, known as MedLitRxSE (17). Building upon Sauceda’s research, Yeh developed the Chinese Medication Literacy Evaluation Scale (ChMLM), a scale specifically designed for adults in Taiwan (18). Lee created a medication literacy survey aimed at adolescents (19). Additionally, Vervloet developed an interview guide to assess medication literacy across three dimensions: the functional domain, the communication domain, and the key domain (20).

Furthermore, numerous scholars have employed and adapted the Risk KAP Survey on Medication Behavior of Chinese Residents, created by the Chinese Pharmacological Society. These surveys employed the KAP model to assess safe medication literacy among residents through the lenses of knowledge, attitude, and behavior (21–27). Nevertheless, a notable absence of universal tools to assess medication literacy among the general public remained, which limited the comprehensive evaluation of medication literacy levels.

The research aimed to develop a model for evaluating the medication safety literacy of community residents, along with a universal assessment scale, with a particular focus on residents of Shenzhen. As a designated special economic zone in China, Shenzhen features a robust economy, a diverse demographic structure, and ample medical resources, reflecting a population that varies in age, educational attainment, and income levels. The Shenzhen municipal government has also committed substantial resources and efforts to enhance drug safety initiatives. Consequently, analyzing the current state of medication literacy among Shenzhen residents is essential, as it provides valuable insights for other cities and regions. This analysis aims to enhance medication literacy among community members, promote the development of high-quality, safe medication practices, and provide evidence-based support for informed policy formulation.

## 2 Materials and methods

### 2.1 Study design and respondents

The research plan received ethical review approval (03, 2024) from the School of Business Administration at Shenyang Pharmaceutical University (Shenyang, China). From June to August 2024, a cross-sectional survey was conducted in the Shenzhen community. This survey employed stratified proportional random sampling methods for data collection. Residents from permanent communities in 11 administrative districts of Shenzhen were selected as representative samples based on economic stratification and proportional sampling by population. Social workers in each administrative district selected eligible residents by random sampling.

The inclusion criteria for participants were: (1) Age≥ 18 years, voluntarily participating in this survey. (2) Residents who have lived in Shenzhen for over 6 months. (3) No history of mental illness and not taking any psychiatric medication. Individuals unable to participate due to hearing, language, cognitive, or other impairments would be excluded from this survey.

The sample size was calculated using the formula $N=\frac{\mu_{\alpha }^{2}\times p(1-p)}{\delta^{2}}\times \text{deff}$ . In the formula, N represents the sample size. A literature review indicated that the prevalence of health literacy among community residents in Shenzhen was estimated to be 47.63% (*p* = 0.4763). The allowable relative error was set at 12%, resulting in an allowable absolute error *δ* = 0.4763 × 12% = 0.057156. The value for μ_α_ was set at 1.96, and the design effect deff was set at 1.5, resulting in a calculated sample size of 439. Considering the ranking of gross domestic product (GDP), the 11 districts of Shenzhen were classified into three tiers. After accounting for an anticipated 15% invalid response rate, the required sample size was adjusted to 1,514 cases.

### 2.2 Survey tool

The research was grounded in the knowledge-attitude-practice (KAP) theory (28) and the conceptual framework of medication literacy (11). It has developed a medication safety literacy assessment model for community residents, focusing on four key dimensions: medication knowledge, medication attitude, medication behaviors, and medication skills. By referencing the KAP assessment scale for the medication behavior risks of Chinese residents published by the Chinese Pharmaceutical Association (21), the 14-item Health Literacy Scale developed by Suka (12), and research by other scholars in the field of medication literacy, and utilized literature research, brainstorming, expert interviews, and the Delphi method to develop and optimize the assessment scale for safe medication literacy among community residents. Additionally, based on age, the assessment scale was divided into a full version and a simplified version: the full version, designed for young adult residents (ages 18–49), consists of 12 sub-dimensions with a total of 34 items; the simplified version, designed for the older adult residents (ages 50 and above), consists of 10 sub-dimensions with a total of 10 items.

The questionnaire consists of three parts: basic information (9 items), a medication literacy assessment scale (Full version for individuals aged 18–49, 34 items; simplified version for individuals aged 50 and above, 10 items), and pharmaceutical service needs (1 item, multiple-choice). The questionnaire demonstrates good reliability and validity, with a Cronbach’s *α* coefficient of 0.924 for the full version and a KMO value of 0.944. For the simplified version, Cronbach’s α coefficient is 0.826, with a KMO value of 0.827, both exceeding 0.7.

The Likert 5 scale was employed for the scoring mechanism. The full version of the scale encompasses 7 items within the dimension of medication knowledge, which are quantitatively assessed with es ranging from 0 to 50, corresponding to a rating of 1 to 5 per item. Other items are scored based on the level of the response, with 4 items related to medication attitude utilizing reverse scoring. The remaining items were scored positively, and all items in the simplified version received positive scores.

Referring to the Chinese health literacy evaluation standards, a score of 80% or above on the questionnaire indicates that community residents possess medication literacy (29). Furthermore, referring to the WHO’s categorization of health literacy levels which was based on the research of Kristine Sørensen (30), medication literacy could be stratified into four levels: ‘failing’ (≤50%), ‘passing’ (>55–66%), ‘good’ (>66–84%), and ‘excellent’ (>84%).

### 2.3 Data collection

Social workers from various administrative regions acted as investigators. They distributed electronic questionnaires to community residents by scanning online codes face-to-face. Investigators assisted older respondents and lower education levels in reading the questionnaire items neutrally and impartially to ensure they understood the questions and answered independently. Investigators meticulously verified the accuracy, completeness, and logical consistency of the questionnaires during data collection. Any responses deemed non-compliant or invalid were excluded to uphold the integrity and reliability of the data. A total of 2,195 questionnaires were collected, of which 2,008 were determined to be valid (1,666 from young adult residents and 342 from older adult residents), while 187 responses were classified as invalid. The valid response rate was 91.48%, with a refusal rate of 8.52%. The online questionnaire was distributed to participants through community service centers in each administrative district, ensuring effective reach to all residents. The analysis demonstrated that the gender and age distribution of the survey participants is consistent with Shenzhen’s demographic data. Chi-square goodness-of-fit tests conducted for jurisdiction, gender, and age reveal that the sample distribution was balanced (*p* > 0.05). The data acquired from the survey were both universal and representative of the population.

### 2.4 Statistical analyses

Data summarization and analysis were performed using Excel 2019, while SPSS 27.0 was employed for statistical evaluations. Socio-demographic data underwent descriptive analysis utilizing frequency counts and proportions. T-tests and F-tests were applied for univariate analyses, contingent upon the data meeting the criteria for normal distribution. If the data did not meet these criteria, non-parametric tests were utilized. Univariate analysis was used to examine the impact of demographic characteristics on medication literacy. Correlation analysis investigated the relationships between medication literacy, knowledge, attitude, behaviors, and skills. Multiple linear stepwise regression was utilized to assess the impact of explicitly correlated independent variables on medication literacy. Structural equation modeling was employed to examine the direct and mediating effects of medication knowledge, attitude, behaviors, skills, and literacy. *p* < 0.05 was considered statistically significant.

## 3 Results

### 3.1 Demographic characteristics

The research included 2,008 residents, of whom 61.7% were male and 38.3% were female. Most participants were aged 18–34 (44.2%). The education level was mainly a bachelor’s degree/junior college (55.1%). The income level was primarily in the range of 5,000–7,999 yuan (44.8%). Furthermore, 42.6% of respondents were involved in medicine field-related work (Table 1).

**Table 1:** Demographic characteristics of community residents (*n* = 2008).

Variable	Group	*n*	%
Gender	Male	1,239	61.7
	Female	769	38.3
Age(years)	18–34	887	44.2
	35–49	779	38.8
	50–64	222	11.1
	≥65	120	6.0
Education	Elementary school and below	58	2.9
	Junior middle school	205	10.2
	Technical secondary school/Vocational high school/High school	533	26.5
	Bachelor/Associate Degree	1,106	55.1
	Master degree or above	106	5.3
Monthly income (yuan)	0–2,999	150	7.5
	3,000–4,999	389	19.4
	5,000–7,999	899	44.8
	8,000–16,999	496	24.7
	≥17,000	74	3.7
Work field	Medical field	855	42.6
	Non-Medical field	1,153	57.4
Health status	Very poor	106	5.3
	Poor	67	3.3
	Commonly	581	28.9
	Good	731	36.4
	Very good	523	26.0
Knowledge of drug categories	Know	1,690	84.2
	Unknow	318	15.8
Pharmaceutical service demand	Low demand	1796	89.6
	High demand	212	10.6

### 3.2 The current status of medication literacy

**Figure 1:**
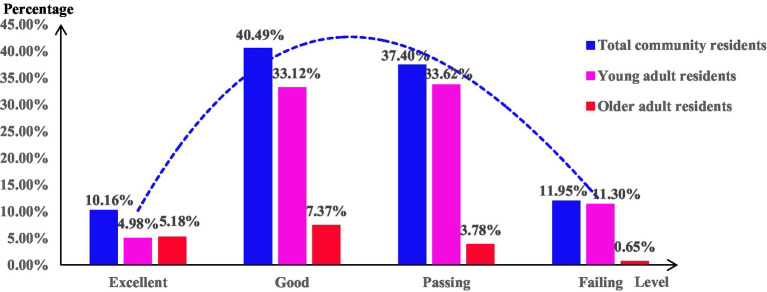
Distribution of medication literacy levels.

A study involving 2,008 community residents comprised 1,666 young adult residents and 342 older adult residents. Referring to the Chinese health literacy evaluation standards, only 12.15% of residents possessed medication literacy. Referring to the World Health Organization’s health literacy standards, the medication literacy among community residents showed a normal distribution: 10.16% reached an excellent level, 40.49% were at a good level, 37.40% were at a passing level, and 11.95% fell into the failing level (Table 2; Figure 1).

**Table 2:** The level of medication literacy.

Category	Percentage (%)		Percentage (%)			
	Equipped	Not equipped	Excellent	Good	Passing	Failing
Young adult residents	9.86	73.11	4.98	33.12	33.62	11.30
Older adult residents	2.29	14.74	5.18	7.37	3.78	0.65
Total community residents	12.15	87.85	10.16	40.49	37.40	11.95

The total medication literacy score among young adult residents was (110.02 ± 21.61), with the mean score for individual items being (3.24 ± 0.64). The standardized mean scores for each dimension of medication literacy, listed from highest to lowest, were as follows: medication skills, medication attitude, medication behaviors, and medication knowledge. Conversely, the total medication literacy score for older adult residents was (31.30 ± 6.92), with the mean score for individual items being (3.13 ± 0.69). The standardized mean scores for each dimension of medication literacy, ranked from highest to lowest, were medication behaviors, skills, knowledge, and attitude (Table 3).

**Table 3:** The score of each dimension.

Category	Dimension	Items	Total score range	Total score	Average score per item	Rank
Young adult residents (*n* = 1,666)	Medication Knowledge	10	10 ~ 50	30.49 ± 10.88	3.05 ± 1.09	4
	Medication attitude	7	7 ~ 35	22.75 ± 5.58	3.25 ± 0.80	2
	Medication behaviors	10	10 ~ 50	31.20 ± 9.61	3.12 ± 0.96	3
	Medication skills	7	7 ~ 35	25.58 ± 5.87	3.65 ± 0.84	1
	Medication literacy	34	34 ~ 170	110.02 ± 21.61	3.24 ± 0.64	
Older adult residents (*n* = 342)	Medication Knowledge	2	2 ~ 10	5.60 ± 2.13	2.80 ± 1.06	3
	Medication attitude	1	1 ~ 5	2.78 ± 1.03	2.78 ± 1.03	4
	Medication behaviors	3	3 ~ 15	10.27 ± 2.67	3.42 ± 0.89	1
	Medication skills	4	4 ~ 20	12.64 ± 3.20	3.16 ± 0.80	2
	Medication literacy	10	10 ~ 50	31.30 ± 6.92	3.13 ± 0.69	

### 3.3 Factors of affect medication literacy

#### 3.3.1 Univariate analysis of influencing factors of medication literacy

The results of the univariate analysis revealed statistically significant differences in medication literacy among community residents, stratified by age, education, income level, field of work, health status, knowledge of drug categories, and pharmaceutical service demand (*p* < 0.001), except for gender. Residents aged 35 to 49 years, possessing a master’s degree or higher, with a monthly income of 17,000 yuan or above, employed in the medical sector, in excellent health, knowledgeable about Rx and OTC medications, and indicating a high demand for pharmaceutical services, tend to exhibit comparatively elevated medication literacy levels (Table 4).

**Table 4:** Univariate analysis of medication literacy.

Items	Group	Medication literacy	t/F	*p*
Gender	Male	3.22 ± 0.64	0.552	0.581	Female	3.21 ± 0.65		
	Age (years)	18–34	3.15 ± 0.63	15.101	<0.001	35–49	3.33 ± 0.63			50–64	3.20 ± 0.69			≥65	3.01 ± 0.69		
Education		Elementary school and below	2.81 ± 0.55	16.556	<0.001	Junior middle school	3.01 ± 0.65			Technical secondary school/Vocational high school/High school	3.17 ± 0.69			Bachelor/Associate Degree	3.28 ± 0.60			Master degree or above	3.37 ± 0.74		
		Monthly income (yuan)	0–2,999	2.97 ± 0.66	11.34	<0.001	3,000–4,999	3.11 ± 0.63			5,000–7,999	3.26 ± 0.63			8,000–16,999	3.29 ± 0.64			≥17,000	3.30 ± 0.74		
			Work field	Medical field	3.34 ± 0.67	7.642	<0.001	Non-medical field	3.12 ± 0.61		
	Health status			Very poor	2.84 ± 0.65	36.037	<0.001	Poor	3.06 ± 0.63			Commonly	3.07 ± 0.56			Good	3.25 ± 0.58			Very good	3.44 ± 0.74		
			Knowledge of drug categories	Know	3.29 ± 0.63	12.131	<0.001
Unknow				2.83 ± 0.59		
Pharmaceutical service demand		Low demand	3.20 ± 0.64	−3.876	<0.001
		High demand	3.38 ± 0.69		

#### 3.3.2 Correlation between variables

The results of the correlation analysis indicated a significant correlation between medication literacy, medication knowledge, attitude, behaviors, and skills, with correlation coefficients ranging from 0.186 to 0.736, all of which were statistically significant (*p* < 0.01) (Table 5).

**Table 5:** The correlation between variables.

Variables	Medication knowledge	Medication attitude	Medication behaviors	Medication skills	Medication literacy
Medication knowledge	1	0.259**	0.310**	0.186**	0.724**
Medication attitude	0.259**	1	0.267**	0.444**	0.591**
Medication behaviors	0.310**	0.267**	1	0.270**	0.736**
Medication skills	0.186**	0.444**	0.270**	1	0.614**
Medication literacy	0.724**	0.591**	0.736**	0.614**	1

^**^p < 0.01.

#### 3.3.3 Multi-factor analysis of influencing factors of medication literacy

The study evaluates collinearity among the independent variables by examining tolerance (TOL) and the variance inflation factor (VIF). A TOL value below 0.1 or a VIF exceeding 10 indicates a considerable collinearity issue among the independent variables. In the research, the TOL values range from 0.722 to 0.863, while the VIF values range from 1.149 to 1.348. It suggests that there are no significant collinearity problems among the independent variables.

Medication literacy scores were employed as the dependent variable. Statistically significant variables (*p* < 0.01) identified in the univariate analysis and medication knowledge, attitude, behaviors, and skills scores were used as independent variables in a stepwise multiple regression analysis. The results showed that the independent variables affecting residents’ medication literacy were, in descending order, medication knowledge, medication behaviors, medication skills, medication attitude, age, education, work field, and income level (Table 6). The result underscored the multidimensional nature of medication literacy. Medication knowledge is fundamental, providing the essential information for appropriate medication use. Conversely, medication attitude is a motivational factor promoting proper medication behaviors. In addition, medication behaviors were a direct manifestation of knowledge and attitude, significantly influencing the safety and effectiveness of medication utilization. Furthermore, medication skills embody the capacity to convert knowledge and attitude into practical behaviors. These four dimensions interact synergistically, collectively shaping the level of medication literacy.

**Table 6:** Influence of different independent variables on medication literacy.

Items	*B*	SE	β	*t*	*p*	Collinearity diagnostics	
						Tolerance	VIF
(constant)	0.066	0.013		5.228	0.000		
Medication behaviors	0.299	0.002	0.442	169.793	0.000	0.8	1.25
Medication knowledge	0.279	0.002	0.469	175.797	0.000	0.761	1.313
Medication skills	0.245	0.002	0.322	117.653	0.000	0.722	1.386
Medication attitude	0.162	0.002	0.215	79.705	0.000	0.742	1.348
Age	0.024	0.002	0.032	12.471	0.000	0.83	1.205
Education	0.009	0.002	0.012	4.367	0.000	0.767	1.303
Work field	−0.016	0.003	−0.012	−4.821	0.000	0.871	1.149
Monthly income	0.004	0.002	0.005	2.157	0.031	0.863	1.159

R^2^ = 0.989, F = 22813.774, *p* < 0.01.

#### 3.3.4 Structural equation modeling analysis of medication literacy

**Figure 2:**
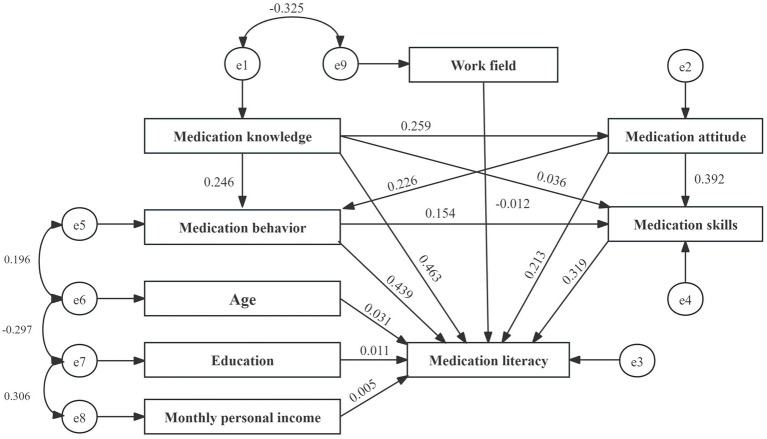
Initial model path and standardized regression coefficients.

**Figure 3:**
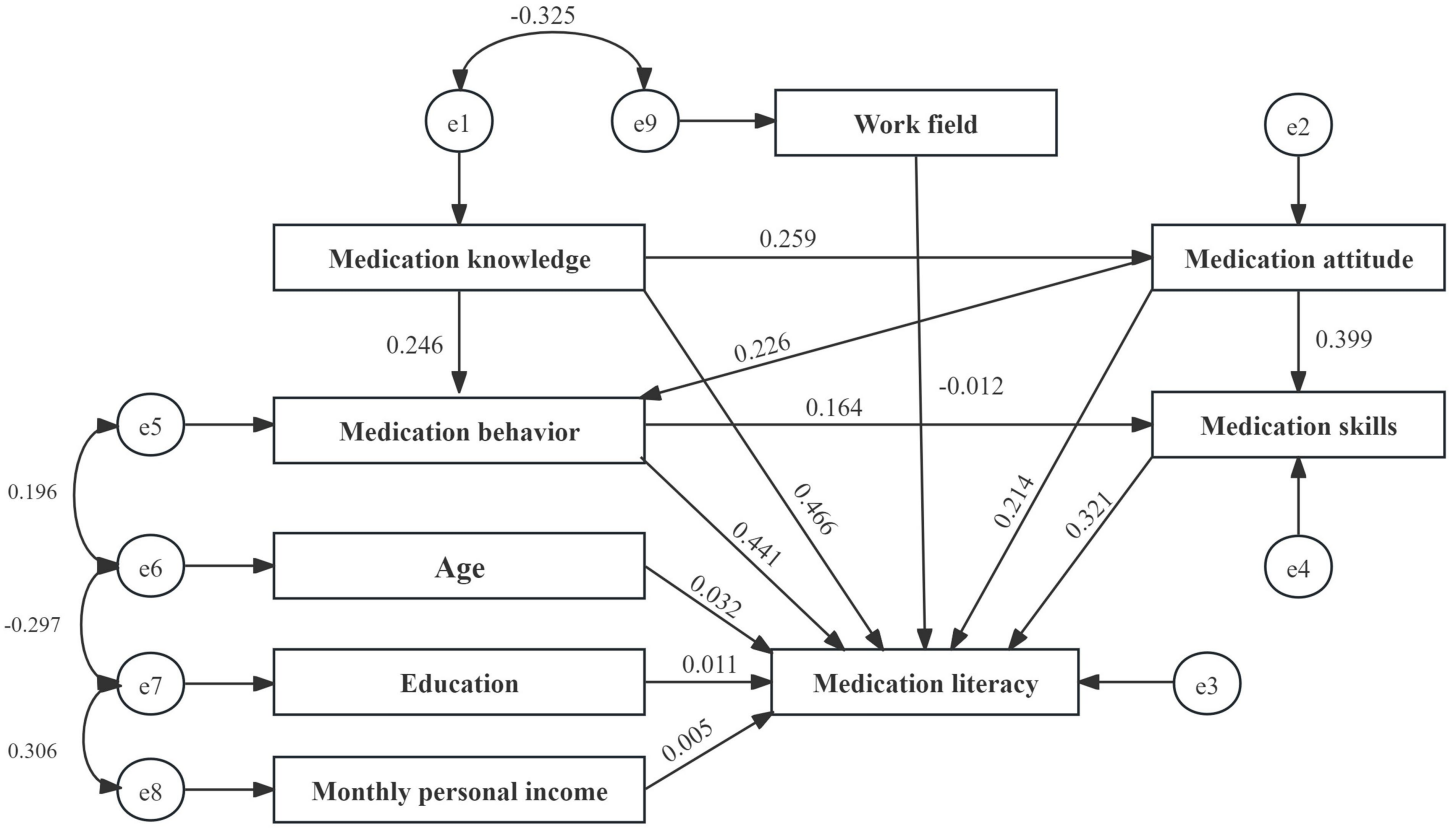
Final model path and standardized regression coefficients.

After constructing the structural equation model, survey data on various variables were integrated. The initial fit results indicated that the model performed well. However, the path coefficient from medication knowledge to medication skills was insignificant (*p* = 0.083). According to the Knowledge-Attitude-Practice (KAP) theory, medication knowledge must be transformed into medication behavior or skills through medication attitudes, which cannot be directly converted into skills, resulting in an insignificant effect. Consequently, the model was modified and re-evaluated, leading to all path coefficients achieving statistical significance (Tables 7, 8; Figures 2, 3).

**Table 7:** Fit indices of the structural equation model for medication literacy.

Fit indices	RMSEA	GFI	NFI	IFI	TLI	CFI
Initial model	0.097	0.963	0.968	0.970	0.939	0.969
Final model	0.094	0.963	0.968	0.969	0.942	0.969

**Table 8:** Path coefficients of the structural equation model for medication literacy.

Path	Standardized path coefficients of the initial model	Standardized path coefficients of the final model
Medication attitude ← Medication knowledge	0.259 (*p* < 0.01)	0.259 (*p* < 0.01)
Medication behaviors← Medication knowledge	0.246 (*p* < 0.01)	0.246 (*p* < 0.01)
Medication behaviors ← Medication attitude	0.226 (*p* < 0.01)	0.226 (*p* < 0.01)
Medication skills ← Medication knowledge	0.036 (*p* = 0.083)	—
Medication skills ← Medication attitude	0.392 (*p* < 0.01)	0.399(*p* < 0.01)
Medication skills ← Medication behaviors	0.154 (*p* < 0.01)	0.164 (*p* < 0.01)
Medication literacy ← Medication knowledge	0.463 (*p* < 0.01)	0.466 (*p* < 0.01)
Medication literacy← Medication attitude	0.213 (*p* < 0.01)	0.214 (*p* < 0.01)
Medication literacy ←Medication behaviors	0.439 (*p* < 0.01)	0.441 (*p* < 0.01)
Medication literacy ←Medication skills	0.319 (*p* < 0.01)	0.321 (*p* < 0.01)
Medication literacy ←Age	0.031 (*p* < 0.01)	0.032 (*p* < 0.01)
Medication literacy ← Education	0.011 (*p* < 0.01)	0.011 (*p* < 0.01)
Medication literacy ← Monthly income	0.005 (*p* = 0.028)	0.005 (*p* = 0.028)
Medication literacy ←Work field	−0.012 (*p* < 0.01)	−0.012 (*p* < 0.01)

The structural equation model revealed that age, education level, income level, and work field moderately influence medication literacy. In contrast, the direct effects of the different dimensions of medication literacy are significant, ranked as follows: medication knowledge, medication behaviors, medication skills, and medication attitude (*β* = 0.466, 0.441, 0.321, and 0.214), with medication knowledge demonstrating the most significant impact.

Moreover, medication knowledge, attitude, and behaviors also significantly affect medication literacy, with indirect and total effect values of 0.239 and 0.705, 0.240 and 0.454, and 0.053 and 0.494. Therefore, enhancing the medication literacy of community residents can be achieved through various strategies, including improving medication knowledge, reinforcing medication attitudes, refining medication behaviors, and developing medication skills. It will help ensure the safe and rational use of medications among community residents.

## 4 Discussion

### 4.1 Current situation of medication literacy among community residents

According to the Chinese health literacy evaluation standards, only 12.15% of residents possessed medication literacy, slightly lower than the findings from other regions in China (31, 32). It may be related to differences in the survey tools and evaluation methods. Previous research has predominantly focused on assessing medication knowledge, attitudes, and behaviors while neglecting to evaluate medication skills. The study was grounded in the knowledge-attitude-practice (KAP) theory and the conceptual framework of medication literacy to incorporate medication skills. It comprehensively evaluates safe medication literacy among community residents across four dimensions: medication knowledge, attitude, behaviors, and skills.

Referring to the World Health Organization’s health literacy standards, the medication literacy among community residents showed a normal distribution. While over half of the residents reached a good level or higher, only 12.15% possessed medication literacy, and merely 10.16% attained an excellent level. These findings indicated that while the overall medication literacy among community residents was relatively ideal, the proportion of residents reaching high levels remained insufficient, suggesting significant room for improvement, especially among those at passing and failing levels. Therefore, the government should continue to encourage community residents to use medications safely and conduct targeted promotions and education on safe medication use to achieve a qualitative leap in the medication literacy level of community residents.

Research findings indicated that residents generally demonstrate an insufficient level of medication knowledge. Among young adult residents, the primary contributing factors to the deficit include a lack of systematic education, reliance on non-professional sources for information acquisition, limited health awareness, and the impact of a fast-paced lifestyle on their learning engagement. In contrast, the lower medication knowledge observed in older adult residents can be primarily attributed to declines in physical, cognitive, and auditory functions, a hesitance to embrace new information, and concerns over the medications’ safety and efficacy, all of which have influenced their beliefs and attitudes toward pharmacological treatments (9).

### 4.2 The associated factors of medication literacy in community residents

The results of the univariate analysis showed that age, education, income level, field of work, health status, knowledge of drug categories, and pharmaceutical service demand were the critical influencing factors of community residents’ medication literacy, consistent with the results of many previous studies (2, 9, 32–37).

The analysis revealed that residents with higher education levels, elevated income, a professional medical background, better health status, excellent knowledge of Rx and OTC medications, and more substantial pharmacy service needs exhibited higher medication literacy scores (Table 4). Notably, residents aged 35–49 possessed higher medication literacy scores than others. It may be attributed to their roles as primary earners within their families, which often entails greater responsibilities and an increased emphasis on safe medication information. Additionally, their extensive life experiences may enhance their capability to recognize and process medicinal details effectively (32, 33). Individuals with a higher level of education present enhanced medication literacy, potentially relevant to their superior ability to acquire and understand information regarding medication usage in their daily lives. Furthermore, they can access a broader range of information channels and possess a more comprehensive knowledge base (34, 35). Likewise, residents with elevated income levels demonstrated improved medication literacy scores, likely due to the enhanced access to quality healthcare resources that higher incomes provide (2, 9). Community members in the medical fields reported higher medication literacy levels than those in non-medical fields. This disparity could be credited to their specialized knowledge and the influence of their work environments.

This study also demonstrates that residents who rated their health status positively tended to attain higher medication literacy scores. Individuals aware of Rx and OTC also displayed superior medication literacy capabilities. Moreover, those experiencing a heightened need for pharmacy services generally achieved higher medication literacy since they benefit from comprehensive resources and information provided by healthcare professionals, augmenting their understanding of medication safety and self-management strategies.

The correlation analysis indicated a positive correlation between medication literacy and knowledge, attitude, behavior, and skills. Medication literacy is related to medication knowledge, attitude, behavior, and skills among residents.

The multifactorial analysis revealed that medication knowledge, attitude, behaviors, and skills positively correlated with medication literacy. Among these variables, medication knowledge significantly influenced medication literacy, followed in descending order by medication behaviors, skills, and attitude. This suggests that a robust comprehension of medication serves as a foundational component. A solid knowledge of medications can foster a positive attitude, encouraging residents to adhere more closely to medical guidance, engage in constructive medication behaviors, and cultivate practical self-management skills. Ultimately, this interrelationship enhances medication safety and efficacy while elevating the overall medication literacy levels within the community. Strengthening various pathways will further contribute to improving medication literacy.

### 4.3 Suggestions for improving medication literacy of community residents

First, several factors influence medication literacy among residents, such as age and education. It was essential to implement tailored educational strategies catering to each demographic’s specific characteristics to enhance medication safety awareness among various age groups within the resident population. Social media platforms and interactive activities can effectively promote medication knowledge for young adult residents. These initiatives can be further enriched by creating question banks and organizing competitive events, fostering engagement and deepening understanding. For older adult residents, effective strategies may include health seminars, group discussions, personalized counseling, educational demonstrations, practical exercises, automated reminders, and the provision of medicine boxes and medication cards. Such comprehensive approaches have the potential to significantly improve residents’ literacy in multiple areas, including medication knowledge, attitude, skills, and behaviors (6). Second, the medication knowledge among residents is relatively weak. By continuously promoting the construction of the Community Drug Safety Service Network Project, educational efforts will be carried out through both online and offline methods. This approach avoids relying solely on traditional classroom teachings, emphasizing innovative promotional methods. Diverse forms of promotion and varied interactive elements aim to enhance medication knowledge among community residents.

## 5 Conclusion

The medication literacy level among community residents was commendable. However, the proportion of individuals exhibiting a high medication literacy level remained insufficient. The analysis of the factors influencing medication literacy indicated that age, education, income levels, occupational fields, health status, knowledge of drug categories, pharmacy service needs, medication knowledge, medication attitude, medication behaviors, and medication skills significantly affect residents’ medication literacy. Enhancing medication literacy among community residents can be accomplished through various strategic approaches. These approaches include improving medication knowledge, reinforcing positive medication attitudes, promoting effective medication-related behaviors, and developing essential medication management skills. By implementing these strategies, the community can facilitate the safe and appropriate use of medications.

### 5.1 Study limitations

It is essential to acknowledge some limitations of this study. First, the study cannot establish causal relationships due to its cross-sectional design. Second, the participants were recruited from the same community, which may restrict the generalizability of the findings. Future research should focus on studying medication literacy across different regions, conducting follow-up assessments to track changes in medication literacy among residents over time, and implementing intervention studies based on our results to enhance medication literacy among residents.

## Data Availability

The original contributions presented in the study are included in the article/supplementary material, further inquiries can be directed to the corresponding authors.
